# Oncological Safety of Intrauterine Manipulator Use in Laparoscopic Hysterectomy for Endometrial Cancer: A Propensity Score-Matched Analysis

**DOI:** 10.3390/medicina61101820

**Published:** 2025-10-11

**Authors:** Yakup Yalcin, Bahadir Kosan, Serenat Yalcin, Kemal Ozerkan

**Affiliations:** Department of Obstetrics and Gynecology, School of Medicine, Bursa Uludag University, Bursa 16110, Turkey; bahadirkosan@uludag.edu.tr (B.K.); serenaty@uludag.edu.tr (S.Y.); ozerkan@uludag.edu.tr (K.O.)

**Keywords:** endometrial cancer, intrauterine manipulator, laparoscopy, recurrence, minimal invasive surgery, overall survival, disease-free survival

## Abstract

*Background and Objectives*: Minimally invasive surgery is considered the standard of care for early-stage endometrial cancer. However, the oncological safety of intrauterine manipulator (IUM) use during laparoscopic hysterectomy remains controversial. The aim of this study was to evaluate the impact of intrauterine manipulator use during laparoscopic hysterectomy on oncological outcomes in patients with clinically early-stage endometrial cancer. *Materials and Methods*: In this retrospective cohort study, 612 patients with FIGO 2009 stage I–III endometrial cancer who underwent staging surgery at a tertiary center between January 2010 and May 2025 were included. Clinical and pathological characteristics were compared between laparoscopy (*n* = 168) and laparotomy (*n* = 444). To reduce selection bias, propensity score matching (PSM) was performed based on age, histological subtype, and FIGO stage. Kaplan–Meier survival analysis and Cox regression modeling were utilized to evaluate disease-free survival (DFS) and overall survival (OS). *Results*: After matching, groups were balanced except for higher rates of para-aortic lymphadenectomy and adjuvant therapy in the laparotomy group. IUM use was not associated with increased LVSI or positive peritoneal cytology. Recurrence was more frequent after laparoscopy (10.1% vs. 6.0%, *p* = 0.028), with inferior 5-year DFS (87.6% vs. 97.4%, HR 5.60, *p* = 0.0006), while OS was similar (82.0% vs. 87.6%, *p* = 0.842). In multivariate Cox analysis, independent predictors of worse DFS were non-endometrioid histology (HR 3.57), FIGO stage III (HR 3.06), grade 3 tumors (HR 2.63), and laparoscopic surgery (HR 0.51). For OS, non-endometrioid histology (HR 5.12), stage III disease (HR 2.98), and grade 3 tumors (HR 4.51) were independent adverse factors, whereas surgical approach was not. *Conclusions*: The use of an intrauterine manipulator in laparoscopic hysterectomy for early-stage endometrial cancer was linked to worse DFS but not OS. These findings suggest caution regarding the routine use of IUMs and highlight the need for prospective randomized trials to clarify their oncological safety.

## 1. Introduction

Endometrial cancer (EC) represents the most frequently diagnosed gynecologic malignancy in developed countries [1]. The standard treatment for early-stage disease is surgical staging, involving total hysterectomy, bilateral salpingo-oophorectomy, and lymph node evaluation [2]. Previous studies have demonstrated that laparoscopic hysterectomy provides oncologic safety comparable to open abdominal hysterectomy, while conferring short-term benefits including reduced postoperative pain, better quality of life, and fewer surgical complications [3,4,5]. In addition, two randomized controlled trials have confirmed the oncologic safety of minimally invasive laparoscopic surgery in endometrial cancer patients [2,6,7].

Intrauterine manipulators (IUMs) are commonly used during laparoscopic hysterectomy to improve surgical performance and outcomes. These instruments facilitate the transection of uterine pedicles, assist in identifying the vaginal fornices, and allow for a safe colpotomy [8]. However, the oncologic safety of IUM use during laparoscopic surgery for endometrial cancer remains controversial [8,9,10,11,12].

The main concerns regarding IUMs center on their potential iatrogenic effects: mechanical pressure may increase the likelihood of lymphovascular space invasion (LVSI), while tumor disruption may promote the spillage of malignant cells through the fallopian tubes or vaginal cuff, with subsequent dissemination into the peritoneal cavity facilitated by CO_2_ insufflation. LVSI, defined as the presence of tumor cells within endothelial-lined spaces of the uterine wall beyond the primary tumor, is a well-established independent adverse prognostic factor in early-stage EC due to its strong association with nodal metastasis and disease recurrence [13,14]. However, Siegenthaler Et Al. reported an 8% rate of peritoneal cytology conversion during laparoscopic surgery with IUM use, a finding linked to unfavorable oncological outcomes [15]. Similarly, other studies have suggested a correlation between IUM use and adverse prognostic features, including LVSI and positive peritoneal cytology [10,11,12,16]. In contrast, several investigations have found no such association, reporting that IUM use during hysterectomy does not affect peritoneal cytology, LVSI, recurrence rates, or survival outcomes [17,18,19,20,21,22].

The debate over IUM use has grown more prominent after the Laparoscopic Approach to Cervical Cancer (LACC) trial, which reported poorer survival with laparoscopy compared to open surgery in cervical cancer patients [9]. More recently, a large multicenter cohort study involving 2661 women across 15 institutions, conducted by Padilla-Iserte Et Al., indicated that the use of an intrauterine manipulator was associated with worse oncologic outcomes in EC patients undergoing minimally invasive surgery [10]. In light of these conflicting results, the oncologic safety of IUMs remains unclear.

The aim of the present study is to investigate whether the use of intrauterine manipulators during laparoscopic surgery for clinically early-stage endometrial cancer has an impact on oncological outcomes, including recurrence patterns, LVSI, peritoneal cytology, disease-free survival (DFS), and overall survival (OS).

## 2. Materials and Methods

This retrospective cohort study was reviewed and approved by the Ethics Committee of Bursa Uludağ University, Faculty of Medicine (Approval No. 2025-08/20). Given its retrospective nature, the need for written informed consent from patients was waived in line with ethical guidelines. The research was carried out in accordance with the Declaration of Helsinki, which provides internationally accepted ethical principles for studies involving human participants.

We retrospectively analyzed the medical records of patients diagnosed with International Federation of Gynecology and Obstetrics (FIGO) stage I–III endometrial cancer who underwent either open or laparoscopic staging surgery at Bursa Uludağ University Hospital between January 2010 and May 2025. The inclusion criteria comprised patients with histopathologically confirmed endometrioid or non-endometrioid endometrial carcinoma, classified as FIGO 2009 stage I–III, who underwent laparoscopic or open hysterectomy with pelvic and/or para-aortic lymphadenectomy. Exclusion criteria were defined as patients with FIGO 2009 stage IV disease, those with concurrent malignancies, or those who had received neoadjuvant therapy. In addition, nine cases in which uterine rupture occurred during insertion of the uterine manipulator or intraoperatively, as well as four patients in whom a manipulator was not used, were excluded from the study.

Medical records were analyzed to collect relevant clinical variables, including demographic data such as age and body mass index (BMI), histopathological characteristics such as histology, disease stage according to the FIGO classification, surgical details including the number of lymph nodes removed and metastatic involvement, and treatment information such as the administration of adjuvant therapy. All surgeries were carried out by gynecologic oncologists, utilizing either an open or laparoscopic technique. Comprehensive surgical staging was performed, encompassing hysterectomy, salpingo-oophorectomy, collection of peritoneal cytology, pelvic and/or para-aortic lymphadenectomy, and omentectomy as required. Para-aortic lymphadenectomy was selectively carried out according to the patient’s risk factors or the surgeon’s discretion. Surgical procedures were performed laparoscopically or via laparotomy. Pelvic lymphadenectomy included removal of the external, internal, and common iliac chains, as well as lymph nodes in the obturator region. Para-aortic dissection was performed to include the area around the vena cava, the aortocaval area and the left para-aortic regions, and up to the level of the renal vein as the upper limit.

Postoperative follow-up was performed every 3–6 months for the first 2 years and every 6 months thereafter until 5 years. Patients who completed 5 years were followed up annually. Further imaging with CT or PET/CT was performed in the presence of clinical findings suggestive of recurrence or metastasis. Findings confirmed by imaging or biopsy according to RECIST 1.1 criteria were considered disease recurrence. Recurrences were classified as local (vaginal, pelvic lymph nodes, bladder, rectum), distant (middle or upper abdominal cavity, para-aortic lymph nodes, lung, liver, bone, brain), or both if present simultaneously. Disease-free survival (DFS) was defined as the time from surgery to disease recurrence or the last follow-up date; overall survival (OS) was defined as the time from surgery to death or the last follow-up date.

Intrauterine manipulators (IUMs) are routinely used in laparoscopic hysterectomy procedures. In a laparoscopic hysterectomy, after the uterine manipulator was inserted but before the initiation of the hysterectomy, both fallopian tubes were coagulated with an energy device. Subsequently, the peritoneal cavity was irrigated with 50 mL of normal saline, and the effluent was collected as intraperitoneal washing fluid for cytological analysis. During the surgery, effective uterine manipulation and optimal exposure of the pelvic cavity were accomplished with the use of a RUMI uterine manipulator (Cooper Surgical Inc., Trumbull, CT, USA).

### Statistical Analysis

All statistical analyses were conducted using IBM SPSS Statistics for Windows, version 26.0 (IBM Corp., Armonk, NY, USA). Continuous variables were compared using the Mann–Whitney U test, while categorical variables were analyzed with the Pearson chi-square test depending on cell frequencies. Continuous data are reported as medians with ranges, and categorical data as counts with percentages. A two-tailed *p*-value of <0.05 was regarded as statistically significant for all analyses. Survival outcomes, namely disease-free survival and overall survival, were evaluated using the Kaplan–Meier method, with differences between survival curves assessed by the log-rank test. To determine independent prognostic factors influencing DFS and OS, Cox proportional hazards regression analyses were conducted.

Propensity score matching (PSM) was utilized to reduce baseline imbalances between the laparoscopic and open surgery groups. Propensity scores were generated through a logistic regression model that included age, histological subtype, and FIGO stage as covariates. Matching between the two groups was performed in a 1:1 ratio using nearest-neighbor matching without replacement, applying a caliper width equal to 0.2 of the standard deviation of the logit of the propensity score. Following matching, standardized mean differences were calculated to evaluate the balance of covariates, with values less than 0.1 considered indicative of adequate balance.

## 3. Results

### 3.1. Patient Characteristics of the Entire Cohort

A total of 612 patients were included in the study (laparoscopy, *n* = 168; laparotomy, *n* = 444) (Table 1). In our study, the median follow-up time was 58.5 months. Several clinicopathological characteristics differed between the groups. Patients undergoing laparoscopy were slightly younger (median 62.0 vs. 63.5 years, *p* = 0.047), had a higher BMI (median 35.0 vs. 33.9 kg/m^2^, *p* = 0.035), lower preoperative CA-125 levels (median 15.0 vs. 17.0 U/mL, *p* = 0.042), and smaller tumor diameters (median 3.5 vs. 4.0 cm, *p* < 0.001). Endometrioid histology predominated in the laparoscopy group, with 92.9% of patients belonging to this subtype. In the laparotomy group, the rate of endometrioid histology decreased to 80.2%, while the rate of aggressive subtypes such as serous (7.9%), clear cell (3.4%), and carcinosarcoma (4.1%) was significantly higher. This suggests that the histological type distribution favors laparoscopy (*p* = 0.004). Similarly, the FIGO stage distribution differed between the two groups. 75.6% of patients in the laparoscopy group were stage IA, while this rate was 60.4% in the laparotomy group. In contrast, the rate of stage III patients was 18.5% in the laparotomy group and only 7.7% in the laparoscopy group. This finding suggests that more advanced-stage cases were significantly more common in patients undergoing laparotomy (*p* = 0.002). A significant difference in tumor grade distribution was observed. In the laparoscopy group, Grade 1, 2, and 3 tumors accounted for 61.3%, 24.4%, and 14.3%, respectively. In contrast, the laparotomy group included 33.8% Grade 1, 33.3% Grade 2, and 32.9% Grade 3 tumors. This difference was statistically significant (*p* < 0.001).

LVSI and depth of myometrial invasion (MI) were less favorable in the laparotomy group (MI, *p* < 0.001; LVSI, *p* = 0.019). There was also a difference in the extent of lymph node dissection: pelvic and para-aortic lymphadenectomy were performed more frequently in the laparotomy group (*p* = 0.003 and *p* < 0.001, respectively), and the number of lymph nodes removed was greater (pelvic, median 22.0 vs. 20.0, *p* = 0.067; para-aortic, median 18.0 vs. 13.0, *p* < 0.001). No difference was observed in peritoneal cytology (*p* = 0.717). Adjuvant treatment patterns differed (*p* < 0.001). Recurrence rates were similar (laparoscopy 10.1% vs. laparotomy 13.1%, *p* = 0.394), and recurrence sites did not differ (*p* = 0.613).

### 3.2. Survival Outcomes of the Entire Cohort

**Figure 1:**
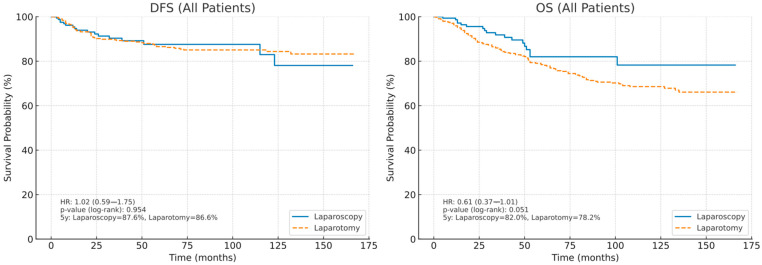
Kaplan–Meier curves of disease-free survival (DFS) and overall survival (OS) in all patients.

In the all-patient analysis, the Kaplan–Meier curves for disease-free survival (DFS) nearly overlapped between laparoscopy and laparotomy, with no evidence of divergence over time (hazard ratio [HR] 1.02, 95% CI 0.59–1.75; log-rank *p* = 0.954). The estimated 5-year DFS was 87.6% after laparoscopy and 86.6% after laparotomy, indicating an absolute difference of 1.0 percentage point. In contrast, the overall survival (OS) curves showed a modest skew in favor of laparoscopy. The risk of death was lower in the laparoscopy group, but statistical significance was borderline (HR 0.61, 95% CI 0.37–1.01; log-rank *p* = 0.051). The estimated 5-year OS was 82.0% after laparoscopy and 78.2% after laparotomy; This corresponded to an absolute difference of 3.8 points. Collectively, these data suggest that laparoscopy was not associated with worse oncologic outcomes in the entire unadjusted cohort and may offer a potential OS advantage over laparotomy, while DFS appears equivalent between the approaches (Figure 1).

### 3.3. Patient Characteristics After Propensity Score Matching

Following propensity score matching, which was conducted using age, histological subtype, and FIGO stage as covariates, two well-balanced groups of 168 patients each were obtained (Table 2). After matching, age, BMI, preoperative CA-125, tumor size, LVSI, depth of myometrial invasion, histological types, FIGO stage, pelvic lymphadenectomy, and peritoneal cytology were similar between the groups (all *p* > 0.05). In the laparoscopy group, Grade 1, 2, and 3 tumors accounted for 61.3% (*n* = 103), 24.4% (*n* = 41), and 14.3% (*n* = 24), respectively. In contrast, in the laparotomy group, the distribution was 42.9% (*n* = 72), 33.9% (*n* = 57), and 23.2% (*n* = 39) for Grades 1, 2, and 3, respectively. This difference between the two groups was statistically significant (*p* = 0.002). Pelvic lymphadenectomy was similar in the extent of lymph node evaluation, but para-aortic lymphadenectomy was still more common in the laparotomy group (74.4% vs. 31.5%, *p* < 0.001), and the number of lymph nodes removed was higher (pelvic, median 27.0% vs. 20.0%, *p* < 0.001; para-aortic, median 18.0% vs. 13.0%, *p* < 0.001). Adjuvant therapy distributions converged but still differed significantly (*p* = 0.013). Peritoneal cytology remained similar (*p* = 0.606). Recurrence was observed in 17 patients (10.1%) in the laparoscopy group compared to 10 patients (6.0%) in the laparotomy group. This difference was statistically significant (*p* = 0.028). Recurrence patterns (local, distant, or both) were also similar (*p* = 0.514).

### 3.4. Survival Outcomes After Propensity Score Matching

**Figure 2:**
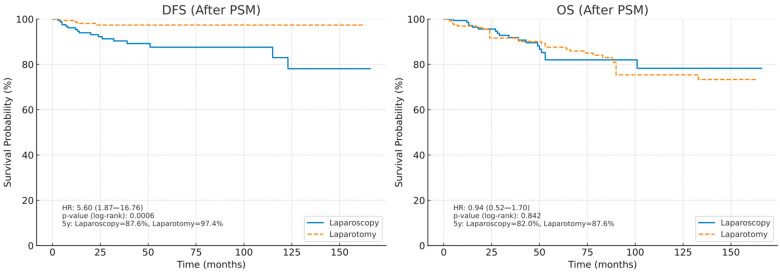
Kaplan–Meier curves of disease-free survival (DFS) and overall survival (OS) in patients after propensity score matching.

By applying propensity score matching with age, histologic subtype, and FIGO stage as covariates, two well-balanced cohorts of 168 patients each were generated, thereby reducing potential baseline differences. Following matching, comparative survival analyses revealed distinct patterns in disease-free survival and overall survival. In terms of DFS, laparoscopy was associated with significantly worse outcomes compared to laparotomy. The hazard ratio (HR) for recurrence in the laparoscopic group was 5.60 (95% CI: 1.87–16.76), indicating a more than fivefold higher risk of recurrence compared to laparotomy. The difference is statistically significant with a log-rank *p*-value of 0.0006. The estimated 5-year DFS rate was 87.6% for patients treated with laparoscopy compared to 97.4% for patients treated with laparotomy, reflecting an absolute difference of approximately 10% in favor of laparotomy. In contrast, OS did not differ significantly between the two groups. The HR for mortality between laparoscopy and laparotomy was 0.94 (95% CI: 0.52–1.70), and a log-rank *p*-value of 0.842 indicates no statistically significant survival advantage for either surgical approach. The 5-year OS estimates were 82.0% for laparoscopy and 87.6% for laparotomy, representing a modest, non-significant difference of 5.6 percentage points (Figure 2).

### 3.5. Univariate and Multivariate Analysis of DFS

In the univariate Cox regression analysis, non-endometrioid histology (HR: 3.10; 95% CI: 1.80–5.35; *p* < 0.001), advanced FIGO stage (Stage III; HR: 2.85; 95% CI: 1.60–5.10; *p* < 0.001), high tumor grade (Grade 3; HR: 2.20; 95% CI: 1.20–4.05; *p* = 0.01), presence of LVSI (HR: 1.50; 95% CI: 1.01–2.25; *p* = 0.04), adjuvant chemotherapy (HR: 2.40; 95% CI: 1.20–4.80; *p* = 0.01), and laparoscopic surgery (Ref: laparotomy; HR: 0.60; 95% CI: 0.40–0.95; *p* = 0.03) were identified as significant prognostic factors for DFS. In the multivariate analysis, independent prognostic factors for DFS included non-endometrioid histology (HR: 3.57; 95% CI: 1.80–7.07; *p* < 0.001), FIGO stage III disease (HR: 3.06; 95% CI: 1.59–5.87; *p* < 0.001), high grade (Grade 3; HR: 2.63; 95% CI: 1.10–6.27; *p* = 0.03), and laparoscopic surgery (HR: 0.51; 95% CI: 0.29–0.92; *p* = 0.03). LVSI, adjuvant treatment subgroups, and other variables did not remain significant in the multivariate model (Table 3).

### 3.6. Univariate and Multivariate Analysis of OS

In the univariate analysis, non-endometrioid histology (HR: 4.50; 95% CI: 2.20–9.10; *p* < 0.001), FIGO stage III disease (HR: 3.00; 95% CI: 1.50–6.00; *p* < 0.001), and high grade (Grade 3; HR: 3.10; 95% CI: 1.50–6.40; *p* < 0.001) were adverse prognostic factors for OS. In the multivariate analysis, independent predictors of OS were non-endometrioid histology (HR: 5.12; 95% CI: 2.26–11.58; *p* < 0.001), FIGO stage III disease (HR: 2.98; 95% CI: 1.41–6.31; *p* < 0.001), and Grade 3 tumors (HR: 4.51; 95% CI: 1.27–15.99; *p* = 0.02). The surgical approach (laparotomy vs. laparoscopy) was not independently associated with OS (Table 4).

## 4. Discussion

In this retrospective study, we assessed the impact of intrauterine manipulator use on oncologic outcomes among patients with clinically early-stage endometrial cancer undergoing minimally invasive management. Analysis of all patients demonstrated that the laparoscopy group was largely composed of patients with earlier disease stages and more favorable histological features, in contrast to the laparotomy group, which included a disproportionately higher number of advanced-stage cases and aggressive histologies. This disparity underscores the strong influence of baseline patient characteristics on surgical approach selection and reflects the inherent risk of selection bias in retrospective analyses.

PSM was applied to control for confounding variables such as age, histological subtype, and FIGO stage, allowing a more balanced comparison between laparoscopy and laparotomy in clinically early-stage endometrial cancer. Following matching, most clinicopathological variables, including age, BMI, tumor size, LVSI, myometrial invasion, histological type, and peritoneal cytology, were comparable between the groups. These findings suggest that the two surgical cohorts were sufficiently harmonized to allow for a more reliable evaluation of oncological outcomes. Despite this balance, certain differences persisted. Grade 1 tumors were more frequently observed in the laparoscopy group, whereas grade 2 and 3 tumors remained more prevalent in the laparotomy group. Notably, para-aortic lymphadenectomy remained significantly more common in the laparotomy group, with a higher number of para-aortic and pelvic lymph nodes retrieved. This is consistent with prior reports demonstrating that open surgery often facilitates more extensive lymphadenectomies, which may reflect both technical feasibility and surgeon preference. Furthermore, differences in adjuvant treatment allocation persisted even after PSM, with more patients receiving adjuvant therapy in the laparotomy group, which may have influenced recurrence and survival outcomes.

Furthermore, Cox regression analysis was performed to account for potential confounding factors. In the univariate analysis, non-endometrioid histology, advanced FIGO stage, high grade, LVSI positivity, adjuvant chemotherapy, and laparoscopy were identified as adverse prognostic factors for DFS. In the multivariate model, independent predictors of DFS included non-endometrioid histology, FIGO stage III disease, grade 3 tumors, and laparoscopic surgery. Regarding OS, univariate analysis demonstrated that non-endometrioid histology, FIGO stage III disease, and grade 3 tumors were adverse prognostic factors, while in the multivariate analysis, the same variables remained independent predictors of OS. In contrast, the surgical approach was not independently associated with OS.

Tohme et al. reported that increased trauma and tumor manipulation during surgery may increase the risk of metastatic disease [23]. The poorer prognosis noted in patients with uterus-confined disease when a uterine manipulator was used supports the hypothesis that the device may disrupt the myometrial barrier, compromise uterine integrity, and consequently worsen oncologic outcomes. Two main hypotheses have been suggested to explain the potential relationship between uterine manipulator use and endometrial cancer dissemination:

1. Macroscopic dissemination. The insertion and manipulation of the device, whether balloon-based or not, may compromise the integrity of the myometrium, leading to iatrogenic uterine rupture and direct exposure of malignant tissue to the peritoneal cavity and surgical field. In addition, tumor fragments shed into the vagina during surgery may potentially spread into the abdominal cavity with gas insufflation following colpotomy.
2. Microscopic dissemination. The use of an IUM markedly increases intrauterine pressure, producing global distension in line with Pascal’s principle. This effect is amplified by the sustained pressure required for uterine mobilization and colpotomy. The elevated intrauterine pressure may facilitate the passive migration of malignant cells through the myometrium into the fallopian tubes and lymphovascular spaces. Such changes in pressure dynamics may also alter the tumor microenvironment, favoring intraoperative hematogenous spread of tumor cells [10].

The use of intrauterine manipulators in endometrial cancer surgery has long been a subject of debate. Some studies have suggested that manipulator use may increase the incidence of lymphovascular space invasion and the rate of positive peritoneal cytology [12,24]. However, other investigations have not confirmed these findings [17,18,19,20,21,22,25]. The current study demonstrated that intrauterine manipulator use after PSM was not associated with a higher rate of either LVSI or peritoneal cytology positivity. This finding supports the existing evidence that neither the surgical approach nor the use of an intrauterine manipulator has a significant impact on the dissemination of malignant cells into the peritoneal cavity.

Janda et al. reported a similar recurrence rate of 7.9% (28/353) following open hysterectomy and 8.1% (33/407) after laparoscopic hysterectomy with intrauterine manipulator use [26]. However, in our study, recurrence was detected in 10 of 168 patients (6.0%) in the laparotomy group and in 17 of 168 patients (10.1%) in the laparoscopy group, and this difference was statistically significant. Although the recurrence patterns were similar, this finding raises concerns that some features of minimally invasive surgery—for example, the use of an intrauterine manipulator or carbon dioxide pneumoperitoneum—may facilitate tumor cell dissemination or implantation. Previous studies have also reported conflicting results on this issue; some reported an increased risk of recurrence with laparoscopic surgery [10], while others did not confirm such an association [27]. Theoretically, tumor cells shed into the vagina could be a risk factor for local recurrence. However, in our study, no difference was found in regional recurrences. Furthermore, this difference remained the same after PSM.

The most recent meta-analysis showed an HR for recurrence of 1.52 (95% CI: 0.99–2.33; *p* = 0.05), suggesting a potential association between intrauterine manipulator use and an increased risk of recurrence. In contrast, no association was found between intrauterine manipulator use and overall survival [10,15]. In our study, disease-free survival and overall survival were similar between laparoscopy and laparotomy in the overall cohort. After propensity score matching, however, DFS was significantly worse in the laparoscopy group (5-year DFS: 87.6% vs. 97.4%), while OS remained comparable between the two groups. Taken together, these results suggest that the use of an intrauterine manipulator during minimally invasive surgery may have a negative impact on DFS but not OS.

One of the strengths of our study is that the long follow-up period of 58.5 months in the 15-year patient data set allows for reliable evaluation of long-term oncological outcomes. Furthermore, propensity score matching was applied for key confounding factors such as age, histological subtype, and FIGO stage, ensuring a more balanced comparison between the laparoscopy and laparotomy groups. The large, single-institution cohort and the availability of detailed clinicopathological data, including tumor grade, stage, LVSI, myometrial invasion, number of lymph nodes, and peritoneal cytology, also represent important strengths of the study.

However, certain limitations should be acknowledged. Due to its retrospective design, the possibility of selection bias could not be completely eliminated. Since cases of laparoscopic surgery performed without a uterine manipulator were not included, the definitive impact of manipulator use on oncological outcomes remains unclear. Future studies comparing laparoscopic hysterectomies performed with and without an intrauterine manipulator (IUM) will allow for more objective and definitive conclusions to be drawn. As this was a retrospective study and only a single cytology sample was obtained from each patient, we were unable to provide information regarding cytology conversion. In addition, the more frequent performance of para-aortic lymphadenectomy and the differences in the distribution of adjuvant therapy in the laparotomy group may have influenced the results. The fact that this study was conducted at a single center may restrict the broader applicability of its findings. Finally, the relatively small number of recurrence and death events may have reduced the statistical power of the multivariable analyses.

## 5. Conclusions

Our results demonstrated that intrauterine manipulator use was not associated with increased rates of positive peritoneal cytology or lymphovascular space invasion. However, recurrence was significantly higher in the laparoscopy group, and disease-free survival was inferior compared with laparotomy, whereas overall survival remained similar. These findings support the ongoing doubts regarding the oncological safety of intrauterine manipulator use during laparoscopic hysterectomy in endometrial cancer patients. In light of these findings, the potential adverse effect of intrauterine manipulators on disease-free survival should be interpreted with caution, and further prospective randomized studies with larger cohorts are warranted to clarify their oncological safety and role in surgical practice.

## Figures and Tables

**Table 1:** Comparison of clinical and pathological characteristics between laparoscopy and laparotomy in the entire patient cohort.

Characteristics	Laparoscopy (*n* = 168)	Laparotomy (*n* = 444)	*p* ^a^ Value
Age, y, median (range), *n*, (%)	62.0 (27.0–85.0)	63.5 (37.0–93.0)	**0.047**
BMI, kg/m^2^, median (range)	35.0 (18.0–54.0)	33.9 (18.4–65.3)	**0.035**
Pre-operative CA-125, (U/mL), median (range)	15.0 (3.0–243.0)	17.0 (2.8–455.0)	**0.042**
Tumor size, cm, median (range)	3.5 (0.2–10.0)	4.0 (0.2–15.3)	**<0.001**
Histological types, *n*, (%)			**0.004**
Endometrioid	156 (92.9%)	356 (80.2%)	
Serous	3 (1.8%)	35 (7.9%)	
Mucinous	0 (0.0%)	6 (1.4%)	
Clear cell	2 (1.2%)	15 (3.4%)	
Carcinosarcoma	4 (2.4%)	18 (4.1%)	
Others ^b^	3 (1.8%)	14 (3.2%)	
FIGO stage ^c^, *n*, (%)			**0.002**
IA	127 (75.6%)	268 (60.4%)	
IB	21 (12.5%)	66 (14.9%)	
II	7 (4.2%)	28 (6.3%)	
III	13 (7.7%)	82 (18.5%)	
FIGO grade			**<0.001**
1	103 (61.3%)	150 (33.8%)	
2	41 (24.4%)	148 (33.3%)	
3	24 (14.3%)	146 (32.9%)	
Depth of myometrial invasion, *n*, (%)			**<0.001**
None	25 (14.9%)	36 (8.1%)	
<50%	115 (68.5%)	278 (62.6%)	
≥50%	28 (16.7%)	130 (29.3%)	
LVSI, *n*, (%)			**0.019**
Absent	137 (81.5%)	319 (71.8%)	
Present	31 (18.5%)	125 (28.2%)	
Pelvic Lymphadenectomy, *n*, (%)			**0.003**
Yes	124 (73.8%)	378 (85.1%)	
No	44 (26.2%)	65 (14.6%)	
Para-aortic Lymphadenectomy, *n*, (%)			**<0.001**
Yes	53 (31.5%)	326 (73.4%)	
No	115 (68.5%)	116 (26.1%)	
Pelvic LN harvested, median (range)	20.0 (2.0–54.0)	22.0 (2.0–82.0)	0.067
Para-aortic LN harvested, median (range)	13.0 (1.0–45.0)	18.0 (1.0–51.0)	**<0.001**
Peritoneal washing cytology, *n*, (%)			0.717
Negative	166 (98.8%)	436 (98.2%)	
Positive	1 (0.6%)	2 (0.5%)	
Unknown	1 (0.6%)	6 (1.4%)	
Adjuvant treatment, *n*, (%)			**<0.001**
None	65 (38.7%)	105 (23.6%)	
Chemotherapy	4 (2.4%)	23 (5.2%)	
Radiotherapy	75 (44.6%)	193 (43.5%)	
Chemotherapy and radiotherapy	24 (14.3%)	123 (27.7%)	
Recurrence, *n*, (%)			0.394
Yes	17 (10.1%)	58 (13.1%)	
No	151 (89.9%)	386 (86.9%)	
Recurrence site, *n*, (%)			0.613
Local	7 (41.1%)	25 (43.1%)	
Distant	6 (35.2%)	27 (46.5%)	
Local+Distant	4 (23.7%)	6 (10.4%)	

BMI: body mass index; CA-125: cancer antigen 125; FIGO: International Federation of Gynecology and Obstetrics; LVSI: lymph-vascular space invasion; LN: Lymph Node; n: number; y: years. Bold values indicate statistical significance. ^a^ *p*-values of age, BMI, CA-125, tumor size, pelvic and para-aortic lymph node harvested were determined by Mann–Whitney U test and the others were determined by the Chi-squared test. ^b^ Villoglandular adenocarcinoma, squamous cell carcinoma, undifferentiated and dedifferentiated carcinoma were included. ^c^ The disease stage was based on the 2009 FIGO staging system.

**Table 2:** Comparison of clinical and pathological characteristics of the cohort after propensity score matching.

Characteristics	Laparoscopy (*n* = 168)	Laparotomy (*n* = 168)	*p* Value ^a^
Age, y, median (range), *n*, (%)	62.0 (27.0–85.0)	63.0 (37.0–85.0)	0.712
BMI, kg/m^2^, median (range)	35.0 (18.0–54.0)	35.5 (18.4–65.3)	0.086
Pre-operative CA-125, (U/mL), median (range)	15.0 (3.0–243.0)	16.0 (2.8–397.0)	0.171
Tumor size, cm, median (range)	3.5 (0.2–10.0)	3.7 (0.5–15.3)	0.643
Histological types, *n*, (%)			0.210
Endometrioid	156 (92.9%)	158 (94.0%)	
Serous	3 (1.8%)	6 (3.6%)	
Mucinous	0 (0.0%)	2 (1.2%)	
Clear cell	2 (1.2%)	0 (0.0%)	
Carcinosarcoma	4 (2.4%)	1 (0.6%)	
Others ^b^	3 (1.8%)	1 (0.6%)	
FIGO stage ^c^, *n*, (%)			0.655
IA	127 (75.6%)	128 (76.2%)	
IB	21 (12.5%)	22 (13.1%)	
II	7 (4.2%)	3 (1.8%)	
III	13 (7.7%)	15 (9%)	
FIGO grade			**0.002**
1	103 (61.3%)	72 (42.9%)	
2	41 (24.4%)	57 (33.9%)	
3	24 (14.3%)	39 (23.2%)	
Depth of myometrial invasion, *n*, (%)			0.064
None	25 (14.9%)	11 (6.5%)	
<50%	115 (68.5%)	114 (67.9%)	
≥50%	28 (16.7%)	43 (25.6%)	
LVSI, *n*, (%)			0.056
Absent	137 (81.5%)	119 (70.8%)	
Present	31 (18.5%)	49 (29.2%)	
Pelvic Lymphadenectomy, *n*, (%)			0.084
Yes	124 (73.8%)	137 (81.5%)	
No	44 (26.2%)	31 (18.5%)	
Para-aortic Lymphadenectomy, *n*, (%)			**<0.001**
Yes	53 (31.5%)	125 (74.4%)	
No	115 (68.5%)	43 (25.6%)	
Pelvic LN harvested, median (range)	20.0 (2.0–54.0)	27.0 (10.0–82.0)	**<0.001**
Para-aortic LN harvested, median (range)	13.0 (1.0–45.0)	18.0 (2.0–44.0)	**<0.001**
Peritoneal washing cytology, *n*, (%)			0.606
Negative	166 (98.8%)	167 (99.4%)	
Positive	1 (0.6%)	0 (0.0%)	
Unknown	1 (0.6%)	1 (0.6%)4%)	
Adjuvant treatment, *n*, (%)			**0.013**
None	65 (38.7%)	41 (24.4%)	
Chemotherapy	4 (2.4%)	4 (2.4%)	
Radiotherapy	75 (44.6%)	104 (61.9%)	
Chemotherapy and radiotherapy	24 (14.3%)	19 (11.3%)	
Recurrence, *n*, (%)			**0.028**
Yes	17 (10.1%)	10 (6.0%)	
No	151 (89.9%)	158 (94.0%)	
Recurrence site, *n*, (%)			0.514
Local	7 (41.1%)	5 (50%)	
Distant	6 (35.2%)	2 (20%)	
Local+Distant	4 (23.7%)	3 (30%)	

BMI: body mass index; CA-125: cancer antigen 125; FIGO: International Federation of Gynecology and Obstetrics; LVSI: lymph-vascular space invasion; LN: Lymph Node; *n:* number; y: years. Bold values indicate statistical significance. ^a^ *p*-values of age, BMI, CA-125, tumor size, pelvic and para-aortic lymph node harvested were determined by Mann–Whitney U test and the others were determined by the Chi-squared test. ^b^ Villoglandular adenocarcinoma, squamous cell carcinoma, undifferentiated and dedifferentiated carcinoma were included. ^c^ The disease stage was based on the 2009 FIGO staging system.

**Table 3:** Univariate and multivariate Cox proportional hazards analysis for disease-free survival (DFS).

Variable	Univariate HR (95% CI)	*p* Value	Multivariate HR (95% CI)	*p* Value
Age	1.01 (0.99–1.04)	0.21	–	–
BMI	1.02 (0.96–1.06)	0.25	–	–
Pre-operative CA-125	1.00 (0.99–1.01)	0.33	–	–
Tumor size	1.03 (0.98–1.08)	0.22	–	–
Histologic type				
Endometrioid	1.00 (–)		–	–
Non-endometrioid	3.10 (1.80–5.35)	**<0.001**	3.57 (1.80–7.07)	**<0.001**
FIGO Stage				
I (Ref.)	1.00 (–)	–	–	–
II	1.40 (0.70–2.80)	0.30	–	–
III	2.85 (1.60–5.10)	**<0.001**	3.06 (1.59–5.87)	**<0.001**
Grade				
1 (Ref.)	1.00 (–)	–	–	–
2	1.65 (0.90–3.01)	0.11	–	–
3	2.20 (1.20–4.05)	**0.01**	2.63 (1.10–6.27)	**0.03**
Positive cytology	0.95 (0.30–3.05)	0.93	–	–
LVSI, Present	1.50 (1.01–2.25)	**0.04**	1.24 (0.72–2.13)	0.44
Depth of myometrial invasion, ≥50	1.15 (0.75–1.75)	0.50	–	–
Methods of surgery, Laparotomy	0.60 (0.40–0.95)	**0.03**	0.51 (0.29–0.92)	**0.03**
Adjuvant treatment				
None (Ref.)	1.00 (–)	–	–	–
Radiotherapy	1.50 (0.80–2.80)	0.20	–	–
Chemotherapy	2.40 (1.20–4.80)	**0.01**	2.69 (0.77–9.35)	0.12
Chemoradiotherapy	1.35 (0.70–2.70)	0.35	–	–

Abbreviations: BMI: Body Mass Index; FIGO: International Federation of Obstetrics and Gynecology; LVSI: Lymphovascular space invasion. Bold values indicate statistical significance.

**Table 4:** Univariate and multivariate Cox proportional hazards analysis for overall survival (OS).

Variable	Univariate HR (95% CI)	*p* Value	Multivariate HR (95% CI)	*p* Value
Age	1.02 (0.99–1.06)	0.18	–	–
BMI	1.01 (0.97–1.08)	0.19	–	–
Pre-operative CA-125	1.00 (0.99–1.01)	0.40	–	–
Tumor size	1.04 (0.98–1.11)	0.20	–	–
Histologic type				
Endometrioid	1.00 (–)		–	–
Non-endometrioid	4.50 (2.20–9.10)	**<0.001**	5.12 (2.26–11.58)	**<0.001**
FIGO Stage				
I (Ref.)	1.00 (–)	–	–	–
II	1.40 (0.60–3.30)	0.40	–	–
III	3.00 (1.50–6.00)	**<0.001**	2.98 (1.41–6.31)	**<0.001**
Grade				
1 (Ref.)	1.00 (–)	–	–	–
2	1.50 (0.70–3.30)	0.29	–	–
3	3.10 (1.50–6.40)	**<0.001**	4.51 (1.27–15.99)	**0.02**
Positive cytology	1.10 (0.30–3.90)	0.90	–	–
LVSI, Present	1.35 (0.75–2.45)	0.31	–	–
Depth of myometrial invasion, ≥50	1.25 (0.70–2.20)	0.45	–	–
Methods of surgery, Laparotomy	0.75 (0.40–1.40)	0.36	–	–
Adjuvant treatment				
None (Ref.)	1.00 (–)	–	–	–
Radiotherapy	1.40 (0.70–3.00)	0.30	–	–
Chemotherapy	1.70 (0.80–3.70)	0.16	–	–
Chemoradiotherapy	1.20 (0.50–2.80)	0.65	–	–

Abbreviations: BMI: Body Mass Index; FIGO: International Federation of Obstetrics and Gynecology; LVSI: Lymphovascular space invasion. Bold values indicate statistical significance.

## Data Availability

The original contributions presented in this study are included in the article. Further inquiries can be directed to the corresponding author.

## Abbreviations

The following abbreviations are used in this manuscript:

BMI	Body Mass Index
CA-125	Cancer antigen 125
CT	Computed tomography
DFS	Disease-free survival
EC	Endometrial cancer
FIGO	International Federation of Obstetrics and Gynecology
IUM	Intrauterine manipulator
LACC	Laparoscopic Approach to Cervical Cancer
LVSI	Lymphovascular space invasion
MI	Myometrial Invasion
OS	Overall survival
PET/CT	Positron emission tomography combined with CT
PSM	Propensity Score Matching
RECIST	Response Evaluation Criteria In Solid Tumors
